# Tunability of topological edge states in germanene at room temperature

**DOI:** 10.1039/d4tc02367f

**Published:** 2024-08-27

**Authors:** Dennis J. Klaassen, Ilias Boutis, Carolien Castenmiller, Pantelis Bampoulis

**Affiliations:** a Physics of Interfaces and Nanomaterials, MESA+ Institute, University of Twente, P.O. Box 217 7500AE Enschede The Netherlands p.bampoulis@utwente.nl

## Abstract

Germanene is a two-dimensional topological insulator with a large topological band gap. For its use in low-energy electronics, such as topological field effect transistors and interconnects, it is essential that its topological edge states remain intact at room temperature. In this study, we examine these properties in germanene using scanning tunneling microscopy and spectroscopy at 300 K and compare the results with data obtained at 77 K. Our findings show that the edge states persist at room temperature, although thermal effects cause smearing of the bulk band gap. Additionally, we demonstrate that, even at room temperature, applying an external perpendicular electric field switches the topological states of germanene off. These findings indicate that germanene's topological properties can be maintained and controlled at room temperature, making it a promising material for low-energy electronic applications.

## Abstract 2

Germanene maintains its topological edge states at room temperature despite thermal smearing of the bulk band gap. These states can be controlled by a perpendicular electric field, highlighting its potential for low-energy electronic applications.

## Graphical abstract

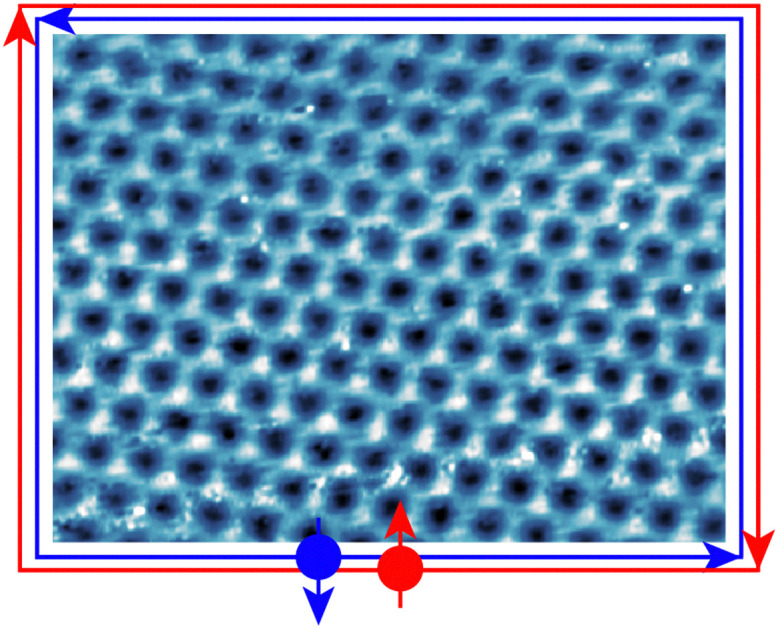

## Introduction

Two-dimensional topological insulators (2DTI) or quantum spin Hall insulators are atomically thin layers characterized by an energy gap in the bulk and two topologically protected gapless helical edge states. Kane and Mele showed that graphene is a 2DTI because of spin–orbit coupling (SOC).^1,2^ SOC induces an ‘effective’ magnetic field that pushes spin-up and spin-down electrons in opposite directions toward the material's edges. Time reversal symmetry prevents backscattering from non-magnetic impurities at these edge states, enabling dissipationless charge transport.^3–8^ However, due to graphene's small SOC-induced gap, observing the QSH effect in graphene requires extremely low temperatures (<0.1 K).^9^ The first practical demonstrations of the QSH effect were in semiconductor quantum wells such as HgTe/(Hg,Cd)Te^6,10^ and InAs/GaSb,^11,12^ and more recently also in other band-inverted ultra-thin materials,^13–15^ adhering to the Bernevig–Hughes–Zhang model.^4^ The challenge at hand is to make a 2DTI with a large band gap, significantly larger than *kT*, such that it still exhibits its topological properties at room temperature^16–18^ in order to harness their properties in device applications, such as topological interconnects, p–n junctions, and field-effect transistors.^19^ Large band gap mono elemental 2DTIs like bismuthene, stanene, and germanene were recently fabricated and could provide a solution to this challenge.^15,20–33^ Recently, topological states have been detected at high temperatures.^34,35^

Germanene is an atomically thin layer of germanium atoms arranged in a buckled honeycomb lattice.^23,36^ Recently, we demonstrated that germanene is a 2DTI with a large enough topological band gap in the order of 70 meV.^37^ The buckled structure of germanene enables us to tune the band gap and the topological state of germanene by applying an electric field perpendicular to the germanene layer.^37–42^ This electric field-induced topological phase transition along with its relatively large band gap makes germanene a candidate for a room-temperature topological field effect transistor.^42^ In this letter, we check the suitability of germanene for room-temperature applications utilizing scanning tunneling microscopy and spectroscopy techniques to study the topological edge states of germanene at room temperature.

## Methods

Scanning tunneling microscopy (STM) and scanning tunneling spectroscopy (STS) measurements are conducted using an ultrahigh vacuum low-temperature scanning tunneling microscope (Omicron LT-STM) operated at 77 K and 300 K using a PtIr tip or Au coated tip. PtIr is chosen for its stability and high conductivity. The tips were prepared by etching high-purity PtIr wire. The Au coating is achieved by doing STM measurements on an Au(111) substrate and gently dipping the tip into the substrate to ensure a thin coating. The background pressure in the ultrahigh vacuum STM chamber is below 3 × 10^−11^ mbar. To grow germanene layers, 1.5 ± 0.5 monolayers of Pt are deposited on an atomically clean Ge(110) substrate. Subsequently, the Ge(110) sample is annealed for several minutes to a temperature of about 1100 K, which is above the eutectic temperature of the PtGe system (1047 K). Above the eutectic point, eutectic droplets are formed on top of the surface with a composition of Pt_0.22_Ge_0.78_. During the cooling process, the sample undergoes spinodal decomposition into a Ge_2_Pt phase and a pure Ge phase.^24,43–47^ During this process, the Ge_2_Pt clusters are decorated with several layers of germanene.^24,37,48,49^ Differential conductivity (d*I*(*V*)/d*V*), was measured at 77 K and room temperature using a lock-in amplifier. The frequency of the lock-in amplifier was set to 1.1–1.2 kHz and the modulation voltage was about 20 mV at 77 K and about 50 mV at room temperature. The spectroscopy data in this paper result from averaging tenths of individual d*I*(*V*)/d*V* curves. The line d*I*(*V*)/d*V* spectroscopy maps in this paper are plotted with a blue-white-red colormap which can be found in ref. 50.

## Results and discussion

**Fig. 1:**
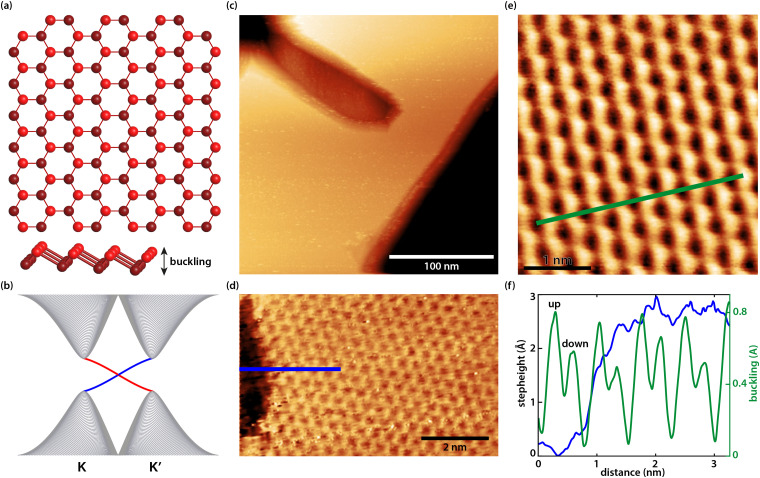
(a) Ball and stick model of the buckled honeycomb lattice of germanene (the bottom image is a side view that shows the buckling effect). (b) A schematic of the band structure of a 2DTI without an external electric field showing the bulk bands at the *K* and *K*′ points of the Brillouin zone (gray) and the edge states filling the bulk gap (blue and red). (c) Large-scale STM image of a Ge_2_Pt cluster covered with germanene. (d) High-resolution STM image on a germanene step edge. (e) Small-scale STM image of the buckled honeycomb lattice of germanene. (f) A graph showing the height profile across the step edge in (d) along the blue line, and a height profile along the green line in (e) revealing the buckling of the germanene lattice.

**Fig. 2:**
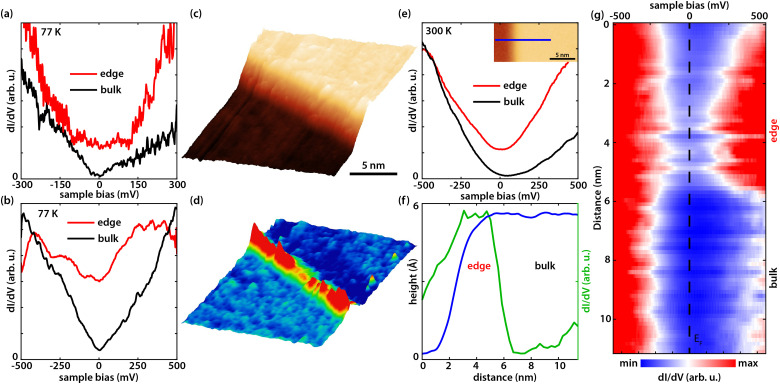
d*I*(*V*)/d*V* point spectra measured at 77 K on the edge and in the bulk of the germanene terrace with an external electric field of (a) ∼1.75 V nm^−1^ and (b) ∼1.95 V nm^−1^. (c) STM image of a germanene step edge and the corresponding d*I*(*V*)/d*V* map in (d) revealing the edge state (0.1 nA, 150 mV). (e) d*I*(*V*)/d*V* point spectrum measured at 300 K with an electric field of ∼1.80 V nm^−1^, on the edge and in the bulk of germanene shown in the inset. (f) Cross-sectional graph showing (blue) the height profile along the blue line in the inset of (e), and (green) the differential conductivity (d*I*(*V*)/d*V*) at the Fermi level (*E*_F_) along the same blue line. The step height is 0.54 ± 0.03 nm, corresponding to the height of two germanene layers. (g) Line d*I*(*V*)/d*V* spectroscopy perpendicular to the edge of the germanene terrace along the blue line in the inset of (e), the location of the edge and the bulk are indicated on the right.

Germanene has a buckled honeycomb structure consisting of two hexagonal sublattices, which are displaced with respect to each other in a direction normal to the germanene layer. In the top view of the ball and stick model of the germanene lattice in Fig. 1(a) the honeycomb structure is visible and the side view reveals the buckling effect. Fig. 1(b) shows a schematic of the band structure at the *K* and *K*′ points of the Brillouin zone of germanene with the edge states (blue and red) filling the bulk band gap. Similar to our earlier work,^37,48^ the first germanene layer (the buffer layer) couples to the underlying Ge_2_Pt(101) substrate causing the layer to be electronically dead. The top germanene layer is a two-dimensional topological insulator that host robust edge states as shown in the schematic of Fig. 1(b).^37^Fig. 1(c) shows a large-scale image of a typical Ge_2_Pt cluster covered with germanene layers including a step edge in the middle of the cluster. A high-resolution STM image on a typical germanene step edge can be seen in Fig. 2(d). Fig. 1(e) reveals a high-resolution STM image of the buckled honeycomb lattice of germanene acquired at room temperature with a lattice constant of (0.42 ± 0.02) nm. From Fig. 1(d) and (e) we can extract some other parameters of the buckled germanene lattice, *i.e.* the buckling and the step height. The graph in Fig. 1(f) shows the height profiles along the blue line in Fig. 1(d) and the green line in Fig. 1(e). From these, we extract a monoatomic step height of about 0.27 ± 0.02 nm, close to the expected 0.28 nm, and a buckling of ∼0.02–0.03 nm. We note here that STM images are influenced by both the topography and the local density of states (LDOS). Because of this, the exact value of the apparent buckling and the step height also depends on sample bias. This explains the slight differences in the measured step height and buckling compared to our previous work.^37^

Fig. 2(a) and (b) show d*I*(*V*)/d*V* point spectra at 77 K at the edge and in the bulk of the germanene terrace at a tip-induced electric field of ∼1.75 V nm^−1^ and ∼ 1.95 V nm^−1^, respectively. In Fig. 2(a) an enhanced differential conductivity is visible at the edge compared to the bulk. Furthermore, the bulk d*I*(*V*)/d*V* curve indicates a band gap. Fig. 2(b) on the other hand reveals a V-shape semi-metallic signature in the bulk with an enhanced differential conductivity at the edges, which is expected for an electric field of ∼1.95 V nm^−1^ (ref. 37) as the tip-induced electric field closes the band gap. The V-shaped density of states (DOS) is one of the hallmarks of a 2D Dirac system. Fig. 2(c) shows a typical STM image on such a germanene edge. Fig. 2(d) shows the corresponding d*I*/d*V* map at the edge state energy revealing the pronounced metallic states at the germanene edge. On top of that, it illustrates that the edge state runs through the whole edge with small changes in intensity, possibly due to reconstructions or local change of termination.^48^ To check the stability of the edge states at room temperature we heated our cryostat to 300 K and repeated the experiments. Fig. 2(e) presents d*I*(*V*)/d*V* point spectra at 300 K at the edge and in the bulk of the germanene layer (shown in the inset) at an electric field of ∼1.80 V nm^−1^. The results in Fig. 2(e) show that the differential conductivity of the edge of the germanene layer is still significantly higher compared to the bulk. Note that the small gap or V-shape cannot be resolved due to thermal broadening of the spectra at room temperature.^51^ The full width at half maximum of the thermal broadening function is equal to 3.5*k*_B_*T*,^48^ which comes down to ∼90 meV at 300 K. On top of that, the instrumental broadening of the lock-in amplifier adds to the total convolution of the d*I*(*V*)/d*V* spectra. The full width at half maximum of the instrumental broadening function is given by 1.7*V*_m_,^48^ where *V*_m_ is the modulation voltage. This means that we have an instrumental broadening term of ∼85 meV at 300 K (*V*_m_ = 50 meV). As a result, the total broadening at full width at half max, given by 
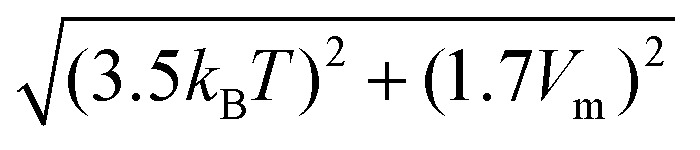
, is ∼124 meV. Therefore, the combination of the thermal broadening and the instrumental broadening convolute the d*I*(*V*)/d*V* signal more than the size of the band gap (∼70 meV) of germanene for small electric fields,^37^ which makes it impossible to resolve the band gap. The blue curve in Fig. 2(f) represents the cross-sectional height profile across the germanene step edge along the blue line in the inset of Fig. 2(e). The height profile, reveals a step height of 0.54 ± 0.03 nm corresponding to about twice the monoatomic step height of germanene. The step height in the inset of Fig. 2(e) therefore translates to the height of two germanene layers on the buffer/Ge_2_Pt(101) substrate. The green curve in Fig. 2(f) reveals the d*I*(*V*)/d*V* cross-section at the Fermi level (*E*_F_) of the d*I*(*V*)/d*V* line spectroscopy measurement in Fig. 2(g), along the same blue line in the inset of Fig. 2(e). From these figures, it becomes clear that the metallic state is present up to room temperature, the edge state decays exponentially into the bulk with the decay length of about 2–3 nm.

**Fig. 3:**
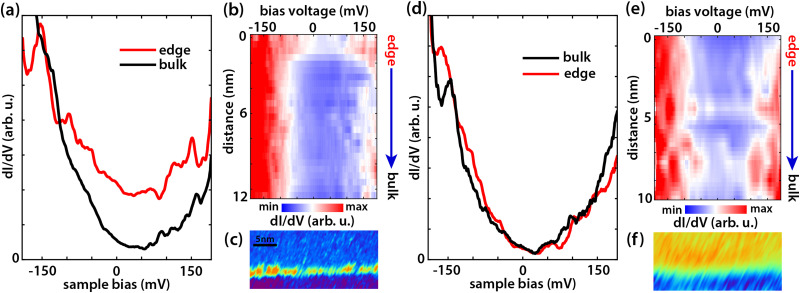
d*I*(*V*)/d*V* point spectra at the edge and in the bulk of the germanene terrace for (a) an electric field below the critical electric field (∼1.80 V nm^−1^) and (d) above the critical electric field (∼2.10 V nm^−1^) at room temperature (300 K). (a) Shows an increased differential conductivity at the edge compared to the bulk, while (d) does not. (b) and (e) Show line d*I*(*V*)/d*V* spectroscopy from the edge towards the bulk of the germanene terrace for (a) an electric field of (∼1.80 V nm^−1^) and (e) for (∼2.10 V nm^−1^). (c) and (f) Represent d*I*(*V*)/d*V* maps of the germanene step edge with setpoints (c) 0.1 nA, −0.1 V (∼1.80 V nm^−1^), and (f) 0.3 nA, −0.5V (∼2.10 V nm^−1^). (c) Shows a state along the step edge of the germanene terrace, while in (f) no state along the step edge can be resolved.

**Fig. 4:**
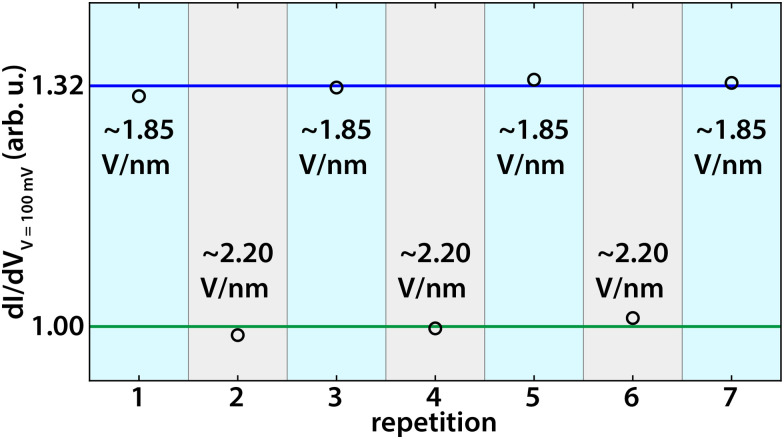
Averaged and normalized (to the 'off' state) d*I*/d*V* recorded at the edge state energy (100 mV) at the germanene step edge for varying tunnel currents/electric fields (black circles). The blue curve represents the average d*I*/d*V* for a tip-induced electric field of ∼1.85 V nm^−1^ when the topological edge state is ‘on.’ The green curve represents the average d*I*/d*V* for a tip-induced electric field of ∼ 2.20 V nm^−1^ when the topological edge state is ‘off’.

Next, we test the effect of the tip-induced electric field on the edge states of germanene. As explained extensively in ref. 37 we apply a local electric field perpendicular to the germanene terrace using the difference in work function between the STM tip and the sample. The electric field can be estimated by *E*_*z*_ = (*Φ*_tip_ − *Φ*_germanene_)/*ez*, where *E*_*z*_ is the electric field, *z* the tip-sample distance, *Φ*_tip_ and *Φ*_germanene_ the work function of the tip (*Φ*_PtIr_ = 5.7 eV and *Φ*_Au_ = 5.2 eV) and that of germanene (*Φ*_Ge_ = 4.0 eV (ref. 52)) respectively, and *e* the elementary charge.^37,53^ Changing the tip-sample separation distance will thus change the local perpendicular electric field. Similarly, we can change the electric field by coating the tip with another material with a different work function. We note here that this method is only an estimate; a systematic error of up to 50% may arise from inaccuracies in the tip-sample distance, work functions, image charges, and band bending,^37^ but does not change the qualitative picture. Increasing the electric field causes charge to shift from one sublattice to the other, breaking inversion symmetry. The band gap closes at a critical field (∼1.95 V nm^−1^) making it a topological semi-metal as demonstrated in our previous work.^37^ Above the critical electric field, the band gap reopens again, the topologically protected edge channels disappear, and the material becomes topologically trivial. With a perpendicular electric field, we can thus change the topological phase of germanene from a topological insulator to a topological semi-metal and then to a trivial band insulator. Fig. 3(a) and (d) present d*I*(*V*)/d*V* point spectra, obtained at room temperature, at the edge and in the bulk of the germanene layer at (a) an electric field below the critical field (∼1.80 V nm^−1^) and (d) at an electric field above the critical field (∼2.10 V nm^−1^). The corresponding d*I*(*V*)/d*V* line spectroscopy measurements are presented in Fig. 3(b) and (e), respectively. Fig. 3(c) and (f) reveal d*I*/d*V* maps on the germanene step edge for a low electric field (∼1.80 V nm^−1^) and a high electric field (∼2.10) V nm^−1^, respectively. The point spectra for an electric field of ∼1.80 V nm^−1^, show that the DOS at the edge is much higher than that at the bulk. The point spectra in Fig. 3(d) at an electric field above the critical electric field show that the state at the edge has disappeared and that the edge and bulk curve almost overlap. Increasing the electric field above the critical electric field (∼1.95 V nm^−1^) switches the edge states of germanene ‘off’ in line with earlier results at 77 K.^37^ For an electric field below the critical electric field, the d*I*(*V*)/d*V* line spectroscopy measurement in Fig. 3(b) shows that the metallic edge state is localized at the edge similar to earlier results in Fig. 2(f) and (g). In the d*I*(*V*)/d*V* line spectroscopy measurement for an electric field above the critical electric field, Fig. 3(e), the state at the edge is no longer present. The same can be seen in the d*I*/d*V* maps in Fig. 3(c) for an electric field of (∼1.80 V nm^−1^) and in Fig. 3(f) for an electric field of (∼2.10 V nm^−1^). Finally, to check the robustness and repeatability of this switching process, we repeated it multiple times. Fig. 4 reveals the results of this experiment. The black circles present the averaged and normalized d*I*/d*V* at the edge state energy at the germanene edge obtained during the repetitions for a tip-induced electric field of ∼1.85 V nm^−1^ where the metallic edge states are ‘on’ and for an electric field of ∼2.20 V nm^−1^ where the metallic edge states are ‘off’. The results show that the switching process of the topological edge channels by applying an external perpendicular electric field is reversible.

## Conclusions

In this study, we have demonstrated that the topological properties of germanene are preserved at room temperature. Using scanning tunneling microscopy and spectroscopy at 300 K, we observed robust metallic edge states in the germanene layer. Although thermal broadening at room temperature prevents us from resolving the band gap, the persistence of edge states confirms the stability of germanene's topological characteristics. This is further supported by the demonstration of the electric-field induced topological phase transition at room temperature, switching the germanene edge states on and off using an electric field. The ability to maintain and control its topological properties under ambient conditions makes germanene a promising candidate for next-generation electronic devices.^19,42,54–57^

## Data availability

Data for this article, including the description of the data are available at 4TU.ResearchData at doi.org/10.4121/6617eb98-b8a0-4676-ae94-1dbb7bb56e02.

## Conflicts of interest

There are no conflicts to declare.

## References

[cit1] Kane C. L., Mele E. J. (2005). Phys. Rev. Lett..

[cit2] Kane C. L., Mele E. J. (2005). Phys. Rev. Lett..

[cit3] Hasan M. Z., Kane C. L. (2010). Rev. Mod. Phys..

[cit4] Bernevig B. A., Hughes T. L., Zhang S.-C. (2006). Science.

[cit5] Bernevig B. A., Zhang S.-C. (2006). Phys. Rev. Lett..

[cit6] König M., Wiedmann S., Brüne C., Roth A., Buhmann H., Molenkamp L. W., Qi X.-L., Zhang S.-C. (2007). Science.

[cit7] Wu C., Bernevig B. A., Zhang S.-C. (2006). Phys. Rev. Lett..

[cit8] Qi X.-L., Zhang S.-C. (2011). Rev. Mod. Phys..

[cit9] Yao Y., Ye F., Qi X.-L., Zhang S.-C., Fang Z. (2007). Phys. Rev. B: Condens. Matter Mater. Phys..

[cit10] Roth A., Brüne C., Buhmann H., Molenkamp L. W., Maciejko J., Qi X.-L., Zhang S.-C. (2009). Science.

[cit11] Knez I., Du R.-R., Sullivan G. (2011). Phys. Rev. Lett..

[cit12] Li T., Wang P., Fu H., Du L., Schreiber K. A., Mu X., Liu X., Sullivan G., Csáthy G. A., Lin X., Du R.-R. (2015). Phys. Rev. Lett..

[cit13] Fei Z., Palomaki T., Wu S., Zhao W., Cai X., Sun B., Nguyen P., Finney J., Xu X., Cobden D. H. (2017). Nat. Phys..

[cit14] Tang S., Zhang C., Wong D., Pedramrazi Z., Tsai H.-Z., Jia C., Moritz B., Claassen M., Ryu H., Kahn S., Jiang J., Yan H., Hashimoto M., Lu D., Moore R. G., Hwang C.-C., Hwang C., Hussain Z., Chen Y., Ugeda M. M., Liu Z., Xie X., Devereaux T. P., Crommie M. F., Mo S.-K., Shen Z.-X. (2017). Nat. Phys..

[cit15] Reis F., Li G., Dudy L., Bauernfeind M., Glass S., Hanke W., Thomale R., Schäfer J., Claessen R. (2017). Science.

[cit16] Xu Y., Yan B., Zhang H.-J., Wang J., Xu G., Tang P., Duan W., Zhang S.-C. (2013). Phys. Rev. Lett..

[cit17] Niu C., Wang H., Mao N., Huang B., Mokrousov Y., Dai Y. (2020). Phys. Rev. Lett..

[cit18] Bai Y., Cai L., Mao N., Li R., Dai Y., Huang B., Niu C. (2022). Phys. Rev. B.

[cit19] Gilbert M. J. (2021). Commun. Phys..

[cit20] Glavin N. R., Rao R., Varshney V., Bianco E., Apte A., Roy A., Ringe E., Ajayan P. M. (2020). Adv. Mater..

[cit21] Matusalem F., Marques M., Teles L. K., Matthes L., Furthmüller J., Bechstedt F. (2019). Phys. Rev. B.

[cit22] Bianco E., Butler S., Jiang S., Restrepo O. D., Windl W., Goldberger J. E. (2013). ACS Nano.

[cit23] Acun A., Zhang L., Bampoulis P., Farmanbar M., van Houselt A., Rudenko A. N., Lingenfelder M., Brocks G., Poelsema B., Katsnelson M. I., Zandvliet H. J. W. (2015). J. Phys.: Condens. Matter.

[cit24] Bampoulis P., Zhang L., Safaei A., van Gastel R., Poelsema B., Zandvliet H. J. W. (2014). J. Phys.: Condens. Matter.

[cit25] Zhang L., Bampoulis P., Rudenko A. N., Yao Q., Van Houselt A., Poelsema B., Katsnelson M. I., Zandvliet H. J. W. (2016). Phys. Rev. Lett..

[cit26] Zandvliet H. J. W. , Xenes, Elsevier, 2022, pp. 27–48

[cit27] Hu T., Hui X., Zhang X., Liu X., Ma D., Wei R., Xu K., Ma F. (2018). J. Phys. Chem. Lett..

[cit28] Yang Z., Wu Z., Lyu Y., Hao J. (2019). InfoMat.

[cit29] Zhao H. , Guo S., Zhong W., Zhang S., Tao L. and Zeng H., Xenes, Elsevier, 2022, pp. 173–196

[cit30] Zhao A. , Xenes, Elsevier, 2022, pp. 49–72

[cit31] Zhu F. F., Chen W. J., Xu Y., Gao C. L., Guan D. D., Liu C. H., Qian D., Zhang S. C., Jia J. F. (2015). Nat. Mater..

[cit32] Deng J., Xia B., Ma X., Chen H., Shan H., Zhai X., Li B., Zhao A., Xu Y., Duan W., Zhang S. C., Wang B., Hou J. G. (2018). Nat. Mater..

[cit33] Bechstedt F., Gori P., Pulci O. (2021). Prog. Surf. Sci..

[cit34] Wu S., Fatemi V., Gibson Q. D., Watanabe K., Taniguchi T., Cava R. J., Jarillo-Herrero P. (2018). Science.

[cit35] Shumiya N., Hossain M. S., Yin J.-X., Wang Z., Litskevich M., Yoon C., Li Y., Yang Y., Jiang Y.-X., Cheng G., Lin Y.-C., Zhang Q., Cheng Z.-J., Cochran T. A., Multer D., Yang X. P., Casas B., Chang T.-R., Neupert T., Yuan Z., Jia S., Lin H., Yao N., Balicas L., Zhang F., Yao Y., Hasan M. Z. (2022). Nat. Mater..

[cit36] Cahangirov S., Topsakal M., Aktürk E., Sahin H., Ciraci S. (2009). Phys. Rev. Lett..

[cit37] Bampoulis P., Castenmiller C., Klaassen D. J., van Mil J., Liu Y., Liu C.-C., Yao Y., Ezawa M., Rudenko A. N., Zandvliet H. J. W. (2023). Phys. Rev. Lett..

[cit38] Ezawa M. (2012). New J. Phys..

[cit39] Ezawa M. (2015). J. Phys. Soc. Jpn..

[cit40] Matthes L., Bechstedt F. (2014). Phys. Rev. B: Condens. Matter Mater. Phys..

[cit41] Vandenberghe W. G., Fischetti M. V. (2017). Nat. Commun..

[cit42] Qian X., Liu J., Fu L., Li J. (2014). Science.

[cit43] Zhang Z., Poelsema B., Zandvliet H. J. W., van Houselt A. (2022). J. Phys. Chem. C.

[cit44] Poelsema B., Zhang Z., Solomon J. S., Zandvliet H. J. W., van Houselt A. (2021). Phys. Rev. Mater..

[cit45] Zhang Z., Poelsema B., Zandvliet H. J. W., van Houselt A. (2021). Phys. Rev. Mater..

[cit46] Poelsema B., Zhang Z., Zandvliet H. J. W., van Houselt A. (2023). Phys. Rev. Lett..

[cit47] van Bremen R., Bampoulis P., Aprojanz J., Smithers M., Poelsema B., Tegenkamp C., Zandvliet H. J. W. (2018). J. Appl. Phys..

[cit48] Zandvliet H. J. W., Klaassen D. J., Bampoulis P. (2024). Phys. Rev. B.

[cit49] Bampoulis P., Castenmiller C., Klaassen D. J., van Mil J., de Boeij P. L., Ezawa M., Zandvliet H. J. W. (2024). 2D Mater..

[cit50] Auton A. , Red Blue Colormap, 2024, https://www.mathworks.com/matlabcentral/fileexchange/25536-red-blue-colormap

[cit51] Walhout C. J., Acun A., Zhang L., Ezawa M., Zandvliet H. J. W. (2016). J. Phys.: Condens. Matter.

[cit52] Borca B., Castenmiller C., Tsvetanova M., Sotthewes K., Rudenko A. N., Zandvliet H. J. W. (2020). 2D Mater..

[cit53] Collins J. L., Tadich A., Wu W., Gomes L. C., Rodrigues J. N. B., Liu C., Hellerstedt J., Ryu H., Tang S., Mo S.-K., Adam S., Yang S. A., Fuhrer M. S., Edmonds M. T. (2018). Nature.

[cit54] Lodge M. S., Yang S. A., Mukherjee S., Weber B. (2021). Adv. Mater..

[cit55] Han W., Otani Y., Maekawa S. (2018). npj Quantum Mater..

[cit56] Ren Y., Qiao Z., Niu Q. (2016). Rep. Prog. Phys..

[cit57] Molle A., Goldberger J., Houssa M., Xu Y., Zhang S.-C., Akinwande D. (2017). Nat. Mater..

